# Characteristics of primary signet ring cell carcinoma of colon and rectum: a case control study

**DOI:** 10.1186/s12876-022-02258-1

**Published:** 2022-04-08

**Authors:** Meng-Tzu Weng, Ko-Han Chao, Chien-Chih Tung, Hao-Chun Chang, I-Lun Shih, Been-Ren Lin, Ming-Jium Shieh, Chia-Tung Shun, Jau-Min Wong, Shu-Chen Wei

**Affiliations:** 1 Department of Internal Medicine, National Taiwan University Hospital and College of Medicine, Taipei City, Taiwan; 2 Department of Internal Medicine, Lo-Sheng Sanatorium and Hospital Ministry of Health and Welfare, New Taipei, Taiwan; 3 Department of Integrated Diagnostics and Therapeutics, National Taiwan University Hospital, Taipei City, Taiwan; 4 Department of Medical Imaging, National Taiwan University Hospital, Taipei City, Taiwan; 5 Department of Surgery, National Taiwan University Hospital, Taipei City, Taiwan; 6 Department of Oncology, National Taiwan University Hospital, Taipei City, Taiwan; 7 Department and Graduate Institute of Forensic Medicine, College of Medicine, National Taiwan University, Taipei, Taiwan; 8 Inflammatory Bowel Disease Clinical and Study Integrated Center, National Taiwan University Hospital, Taipei, Taiwan

**Keywords:** Colorectal cancer, Primary signet ring cell carcinoma, Young patients

## Abstract

**Background:**

Primary signet ring cell carcinoma of the colon and rectum (PSRCCR) is rare, usually diagnosed at advanced stage with poor outcomes. We aimed to find possible diagnostic clues in order to help diagnosis.

**Methods:**

A retrospective study of PSRCCR patients from 1993 to 2018 was reviewed at a single tertiary center. Colorectal adenocarcinoma patients as control group with 1:4 ratio was also enrolled.

**Results:**

18 patients with PSRCCR were identified. The prevalence rate was 0.16% (18 of 11,515). The mean age was 50.2 years-old in PSRCCR group and 63 years-old in non-SRCC colorectal cancer patients (*p* < 0.001). Diagnosis tool depends on colonoscopy were much less in PSRCCR group than control group (44.4% vs 93%, *p* < 0.001). SRCC patients had higher level of CEA (68.3 vs 17.7 ng/mL, *p* = 0.004) and lower level of Albumin (3.4 vs 4.3 g/dL, *p* < 0.001). The majority of PSRCCR tumor configuration was ulcerative and infiltrative. More PSRCCR pathology presented as high-grade carcinoma (66.7 vs 1.4%, *p* < 0.001) and lymphovascular invasion (77.8 vs 44.4%, *p* = 0.011) than control group. More PSRCCR patients were diagnosed at advanced stage (88.8 vs 40.3%, *p* = 0.001). Higher mortality was also noticed in PSRCCR group than control group (72.2 vs 20.8%, *p* < 0.001).

**Conclusion:**

For young patients with long segment colonic stenosis and ulcerative/ infiltrative mucosa but endoscopic biopsy failed to identify malignant cells, earlier operation or non-colon site biopsy is suggested for diagnosing the PSRCCR.

## Background

Primary signet ring cell carcinoma of the colon and rectum (PSRCCR) is a rare histologic subtype, accounting for approximately 0.6–2.7% of all adenocarcinomas of the colon [1, 2]. Signet ring cell carcinoma (SRCC) contains a large amount of mucin, which pushes the nucleus to the cell periphery. The World Health Organization has a specific criterion for diagnosing this subtype; that is, the presence of > 50% of signet cells [3]. The symptoms of PSRCCR include bloody stool, body weight loss, abdominal pain or bowel habit change and usually appear at late stage [4]. The symptoms are similar to those of colorectal cancer (CRC) [5], whereas the clinical behavior is more aggressive than colorectal cancer. Patient with colonic signet ring cell carcinoma were more frequently diagnosed at advanced stage (75.2–91%) [6–8] than patients with colon adenocarcinoma (43.6–48%). Less than 40% of cases have a change to receive curative operation [9]. Compared with non-SRCC colorectal cancer, PSRCCR tends to occur at a younger age, presented as scirrhous appearance, with more lymphovascular and peritoneal involvement, and has a poorer prognosis [6, 10, 11].

As PSRCCR is a rare subtype and its characteristic is different from common colon adenocarcinoma. In this study, we aimed to compare the difference of clinical characteristics, pathologic features, diagnostic stage and outcome between patients with SRCC and non- SRCC colorectal cancer. Through this comparison, we also would like to find the possible diagnostic clues in order to help diagnosis.

## Methods

### Data collections

This is a retrospective study reviewed the colorectal cancer patients from October 1993 to June 2018 in National Taiwan University Hospital. In total, 11,515 colorectal cancer tissues were received and registered in the pathology databank. Clinical information including demographic data, laboratory, endoscopic and pathologic report, treatment regiments and the disease course were assessed. The pathological diagnosis of SRCC was confirmed through histological examination (using hematoxylin and eosin staining) which revealed the presence of signet ring cell was greater than 50%. Tumor configuration was classified into 3 types: exophytic, ulcerative and infiltrative. Exophytic is defined as an abnormal growth that sticks out from the surface of a tissue. Ulcerative is defined as the lesion is depressed than surrounding mucosa. Infiltrative is defined as the margin between cancerous tissue and surrounding non-cancerous tissue is poorly demarcated. In Crohn’s disease, stricture ≤ 5 cm is defined as short stricture [12]. In this study, we defined wall thickening > 5 cm as long segment. Tumor stage was based on TNM staging system and American Joint Committee on Cancer, AJCC Cancer Staging Manual (8th edition, 2017). Stage I and II were defined as early stage and stage III and IV as advanced stage. The survival duration was based on the last outpatient department date or date of death. A retrospective computer-aided search generated 18 PSRCCR cases. In order to compare the stage, location of tumor, age, sex, also omitting the outcomes influenced by the treatment choices in different year, we match PSRCCR patients with non-PSRCCR colorectal cancer patients as a control group at a 1: 4 ratio according to the year of patient diagnosed with colorectal cancer. This study was approved by the Ethics Review Board of National Taiwan University Hospital (IRB Number 201808070RINB). The Institutional Review Board of NTUH allowed to waive the informed consent because of the retrospective nature of the study and the analysis used anonymous clinical data.

### Statistical analyses

Results are expressed as the mean and range. Continuous variables were expressed as mean ± standard deviation (SD). Categorical variables were expressed as frequency (percentage). Student’s t-test was used for quantitative variables and Chi-square statistic was used for categorical variables among the two cohorts. A p-value less than 0.05 was considered as statistically significant. The survival was calculated using the Kaplan–Meier method. Prognostic factors including age, sex, underlying disease, laboratory data, tumor subtype, pathological parameters, cancer stage were included in survival analyses. Parameters with *p* < 0.05 in univariable analyses were further checked by multivariable Cox proportional hazard model. These analyses were carried out with SPSS 11.0 program (SPSS, Paris, France).

## Results

### Demographic and clinical characteristics of PSRCCR patients

A total of 11,515 patients were diagnosed with colorectal cancer from the hospital database between 1993 and 2018. Among them, 18 were identified with PSRCCR. The incidence of PSRCCR was 0.16% in colorectal patients. A total of 72 patients with non-SRCC CRC were included as controls. PSRCCR patients was significant younger than non-SRCC CRC patients (mean age 50.2 vs 63 years-old, *p* < 0.001). In both the PSRCCR and non- SRCC groups, male predominance was noted (66.7% vs 62.5%, *p* = 0.74). The baseline hypertension (22.2% vs. 38.9%, *p* = 0.19), diabetes mellitus (5.6% vs. 16.7%, *p* = 0.23), hyperlipidemia (0% vs. 12.5%, *p* = 0.20), viral hepatitis (5.6% vs. 5.6%, *p* = 1), coronary artery disease (5.6% vs. 11.1%, *p* = 0.48) and chronic kidney disease (5.6% vs. 9.7%, *p* = 0.58), was comparable in these two groups. Most of the non-SRCC CRC patients were diagnosed by colonoscopy. In contrast, more than one-third PSRCCR patients were diagnosed by operation or non-colon site biopsy (*p* < 0.001). The PSRCCR group was associated with higher level of CEA (68.3 vs 17.7 ng/mL, *p* = 0.004), the albumin level was significantly lower in the PSRCCR group than in the non-SRCC group (3.4 *vs* 4.3 g/dL, *p* < 0.001). The clinical information of PSRCCR and non-SRCC CRC patients were summarized in Table 1. We further compared the CEA and Albumin level between tumor stage III and IV and noticed that the CEA level was significantly higher in stage IV than stage III (124.9 vs 7.66, *p* = 007). There was no significant difference of albumin level between stage IV and III (4.13 vs 4.3, *p* = 1).

**Table 1:** Clinical characteristics of colorectal cancer patients

	SRCC (n = 18)	Non-SRCC (n = 72)	*p* value
Demographics			
Age, mean (25–75 th)	50.2 (29.5–67.5)	63.0 (54.5–72.5)	< 0.001
Male sex (%)	12 (66.7)	45 (62.5)	0.74
Underlying diseases, n (%)			
Hypertension	4 (22.2)	28 (38.9)	0.19
Diabetes Mellitus	1 (5.6)	12 (16.7)	0.23
Hyperlipidemia	0 (0)	9 (12.5)	0.20
Viral hepatitis	1 (5.6)	4 (5.6)	1
Coronary artery disease	1 (5.6)	8 (11.1)	0.48
Chronic kidney disease	1 (5.6)	7 (9.7)	0.58
Diagnosis tool			0.001
Colonoscopy	9 (50)	67 (93.1)	
Operation or non-colon biopsy	9 (50)	5 (6.9)	
Laboratory			
CEA (ng/mL)	68.3	17.7	0.004
WBC (K/μL)	7485	7387	0.83
Hb (g/dL)	12.3	12.3	0.26
Alb (g/dL)	3.4	4.3	< 0.001
ALT (U/L)	20.4	25.3	0.51
Cre (mg/dL)	0.88	1.07	0.41

Student’s t-test was used for quantitative variables and Chi-square statistic was used for categorical variables among the two cohorts

CEA, Carcinoembryonic antigen; WBC, White blood cell; Hb, Hemoglobin; Alb, Albumin; ALT, alanine aminotransferase; Cre, Creatinine

### Comparison of pathologic characteristics between PSRCCR and non- SRCC CRC patients

Majority of the tumor were located at left side in both groups (61.1% vs 68.1%, *p* = 0.59). Most tumor configuration of PSRCCR patients were ulcerating or infiltrative, whereas those of non-SRCC patients were exophytic or ulcerating (*p* < 0.001). The differentiation grade of PSRCCR group was significantly advanced than that of non-SRCC group (66.7% vs 1.4% high grade, *p* < 0.001). PSRCCR patients also had more lymphovascular invasion than non-SRCC patients (77.8% vs 44.4%, *p* = 0.01). The pathologic features were listed in Table 2.

**Table 2:** Pathologic characteristics of colorectal cancer patients

	SRCC (n = 18)	Non-SRCC (n = 72)	*p* value
Tumor location			0.59
Right side	7 (38.9)	23 (31.9)	
Left side	11 (61.1)	49 (68.1)	
Tumor configuration			< 0.001
Exophytic	3 (17.6)	34 (47.2)	
Ulcerating	7 (41.2)	35 (48.6)	
Infiltrative	7 (41.2)	3 (4.2)	
Grade			< 0.001
Low	1 (5.6)	69 (95.8)	
High	12 (66.7)	1 (1.4)	
Non-applicable	5 (27.7)	2 (2.8)	
Lymphovascular invasion	14 (77.8)	32 (44.4)	0.01
Surgical margin involvement	1 (5.6)	3 (4.2)	0.60

Chi-square statistic was used for categorical variables among the two cohorts

### Comparison of tumor stage between PSRCCR and non-SRCC CRC patients

**Fig. 1:**
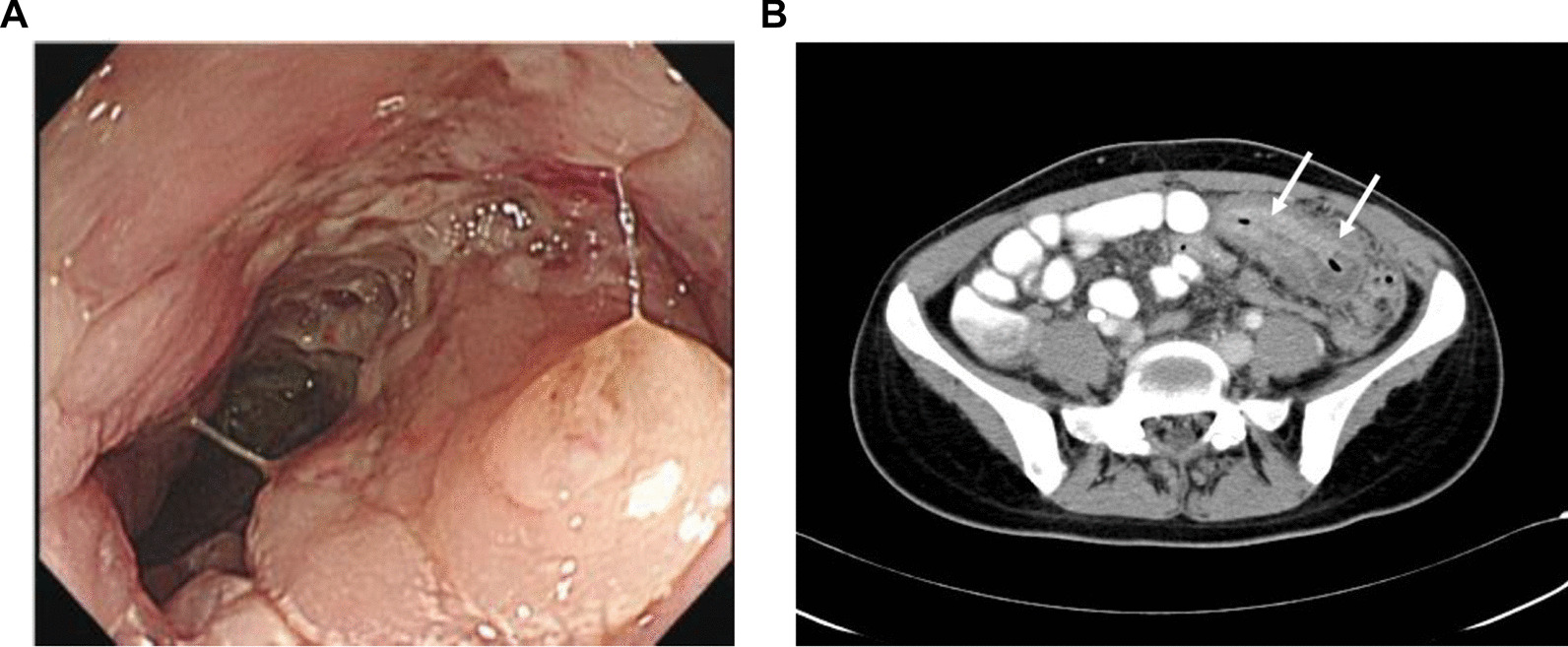
Typical endoscopic and CT image of SRCC. **A** The colonoscopy revealed edematous fragile mucosa with ulcer that led to lumen stenosis at transverse colon. **B** Abdominal computer tomography (CT) showed segmental wall-thickening of transverse colon with increased enhancement and prominent adjacent fat-stranding (arrow)

Most of SRCC patients were diagnosed at stage T3 or T4 (94.4%) and N2 (77.8%). The distant metastasis rate was 50%. The only patient who was diagnosed with a tumor at an early stage, with carcinoma in situ, was due to a positive immunochemical fecal occult blood test during health examination. The number of the patients with initial AJCC stages 0 and 1, 2, 3, and 4 was 1 (5.6%) versus 0 (0%), 0 (0%) versus 23 (31.9%), 1 (5.6%) versus 20 (27.8%), 9 (50%) versus 18 (25%) and 7 (38.8%) versus 11 (15.3%) in the PSRCCR and non-SRCC groups, respectively (*p* = 0.001). All these CRC patients underwent operation. As most PSRCCR patients were diagnosed at advanced stage, 88.9% of them also received chemotherapy or combine therapy. In contrast, a curative resection (R0 resection with related radical lymphatic dissection) was performed in 37 (51.4%) non-SRCC patients. The PSRCCR group had shorter follow-up period than non-SRCC group (15 vs 94.5 months, *p* < 0.001) (Table 3). The clinical characteristic, colonoscopic and histologic finding of the 18 PSRCCR patients were listed in Table 4. Typical endoscopic and CT images (case 18) was shown in Fig. 1. Seventeen PSRCCR patients with initial CT were reviewed. Most of the patients (13 of 17; 76.5%) presented with long segmental wall thickening and increased enhancement of the involved colon. The average length of the thickened wall was 6.6 cm (range 4.4–11.6 cm). Only 3 of the 15 (20%) patients presented with an intraluminal mass by colonoscopy. One patient (5.9%) had carcinoma in situ, which could not be identified on CT.

**Table 3:** Treatment and tumor stage of colorectal cancer patients

	SRCC (n = 18)	Non-SRCC (n = 72)	*p*-value
Treatment			
Operation	2 (11.1)	37 (51.4)	0.001
Op + C/T	4 (22.2)	20 (27.8)	
Op + C/T + Target	10 (55.6)	11 (15.3)	
Op + CCRT	2 (11.1)	4 (5.6)	
T			0.002
Tis	1 (5.6)	0	
1	0	4 (5.6)	
2	0	22 (30.5)	
3	8 (44.4)	36 (50)	
4	9 (50)	10 (13.9)	
N			< 0.001
0	2 (11.1)	44 (61.1)	
1	2 (11.1)	21 (29.2)	
2	14 (77.8)	7 (9.7)	
M			0.002
0	9 (50)	62 (86.1)	
1	9 (50)	10 (13.9)	
Stage			0.001
0	1 (5.6)	0	
1	0	23 (31.9)	
2	1 (5.6)	20 (27.8)	
3	9 (50)	18 (25)	
4	7 (38.8)	11 (15.3)	
Follow-up duration, mean (months)	15	94.5	< 0.001

Chi-square statistic was used for categorical variables among the two cohorts

Op, operation; CT, Chemotherapy; CCRT, concurrent chemoradiotherapy

**Table 4:** Clinical characteristics of SRCC patients

No	Age	Sex	Symptoms	CT Findings	Colonoscopic finding	Histology configuration	Diagnosis tool	Cancer stage	FU (m)	Survival
1	36	M	Abdominal fullness	Bowel wall thickening Length: from transverse to ascending colon	Incomplete study due to patient intolerance	not mentioned	Colonoscopy	T4bN2bM1c	9	Loss FU
2	74	M	Bloody stool	Bowel wall thickening with serosal invasion Length: 5.8 cm	A cauliflower-like tumor with central ulceration at ascending colon	Infiltrative	Colonoscopy	T4aN2bM1c	23	no
3	86	F	Colon obstruction	Bowel wall thickening Length: 6 cm	A cauliflower-like tumor at cecum	Ulcerative	Colonoscopy	T4aN2bM1a	1	Loss FU
4	28	F	Bloody stool	Not performed	An ulcerative tumor with easily bleeding at sigmoid colon	Ulcerative	Colonoscopy	T3N2bM0	13	no
5	45	F	Noted during hemorrhoidectomy	Semi-circumferential mass	Not performed	Ulcerative	Operation	T4N2bM0	19	Loss FU
6	14	M	Chronic diarrhea	Bowel wall thickening and ascites Length: 11.6 cm	Colon ulcers with lumen stenosis at sigmoid colon	Ulcerative	Colonoscopy	T4bN2bM1c	17	no
7	17	M	Bloody stool	Bowel wall thickening Length: 7 cm	Erosive and fragile mucosa with luminal stenosis at sigmoid colon	Infiltrative	Skin biopsy	T3N2bM1b	14	no
8	76	M	Bloody stool	Bowel wall thickening Length: 5.2 cm	Infiltrative tumor at rectum	Infiltrative	Operation	T3N0M0	37	no
9	61	M	Positive stool occult blood	Negative finding	A semi-annular tumor at sigmoid colon	Ulcerative	Colonoscopy	TisN0M0	100	yes
10	31	M	Abdominal fullness	Bowel wall thickening Length: 5.5 cm	Infiltrative tumor with lumen stenosis at descending colon	Infiltrative	Operation	T4aN2bM1c	44	Loss FU
11	76	M	Right lower limb edema	Bowel wall thickening Length: 5.2 cm	A circular ulcerative tumor at S-D junction	Exophytic	Abdominal LN biopsy	T3N2bM0	15	no
12	78	M	Constipation	Bowel wall thickening Length: 6 cm	An ulcerative tumor with luminal stenosis at sigmoid colon	Ulcerative	Colonoscopy	T3N2bM0	8	no
13	40	F	Abdominal fullness	Bowel wall thickening and regional LAP Length: 8.2 cm	A cauliflower like tumor with luminal obstruction at splenic flexure	Exophytic	Colonoscopy	T4bN2bM1c	10	no
14	39	M	Anemia	Bowel wall thickening Length: 6.9 cm	Chicken skin change and congestion of mucosa at hepatic flexure	Infiltrative	Peritoneal biopsy	T3N2aM1C	14	no
15	57	F	Anemia	Bowel wall thickening and regional LAP Length: 7.4 cm	Multiple lobulated tumors at sigmoid colon	Infiltrative	Colonoscopy	T3N2bM0	17	no
16	85	M	Colon perforation	Bowel wall thickening and pneumoperitoneum Length: 4.4 cm	Not performed. Ascending colon perforation	Ulcerative	Operation	T4aN1bM0	2	no
17	43	M	Incidental finding by CT	Intraluminal mass	Not performed	Exophytic	Operation	T3N1bM0	28	no
18	18	F	Colon obstruction	Bowel wall thickening and abscess Length: 6.8 cm	Erosive and fragile mucosa with lumen stenosis at transverse colon	Infiltrative	Operation	T4aN2bM1c	15	no

CT, Computed Tomography; LA P, Lymphadenopathy; FU, follow-up; m, month

### Comparison of survival status between PSRCCR and non-SRCC CRC patients

**Fig. 2:**
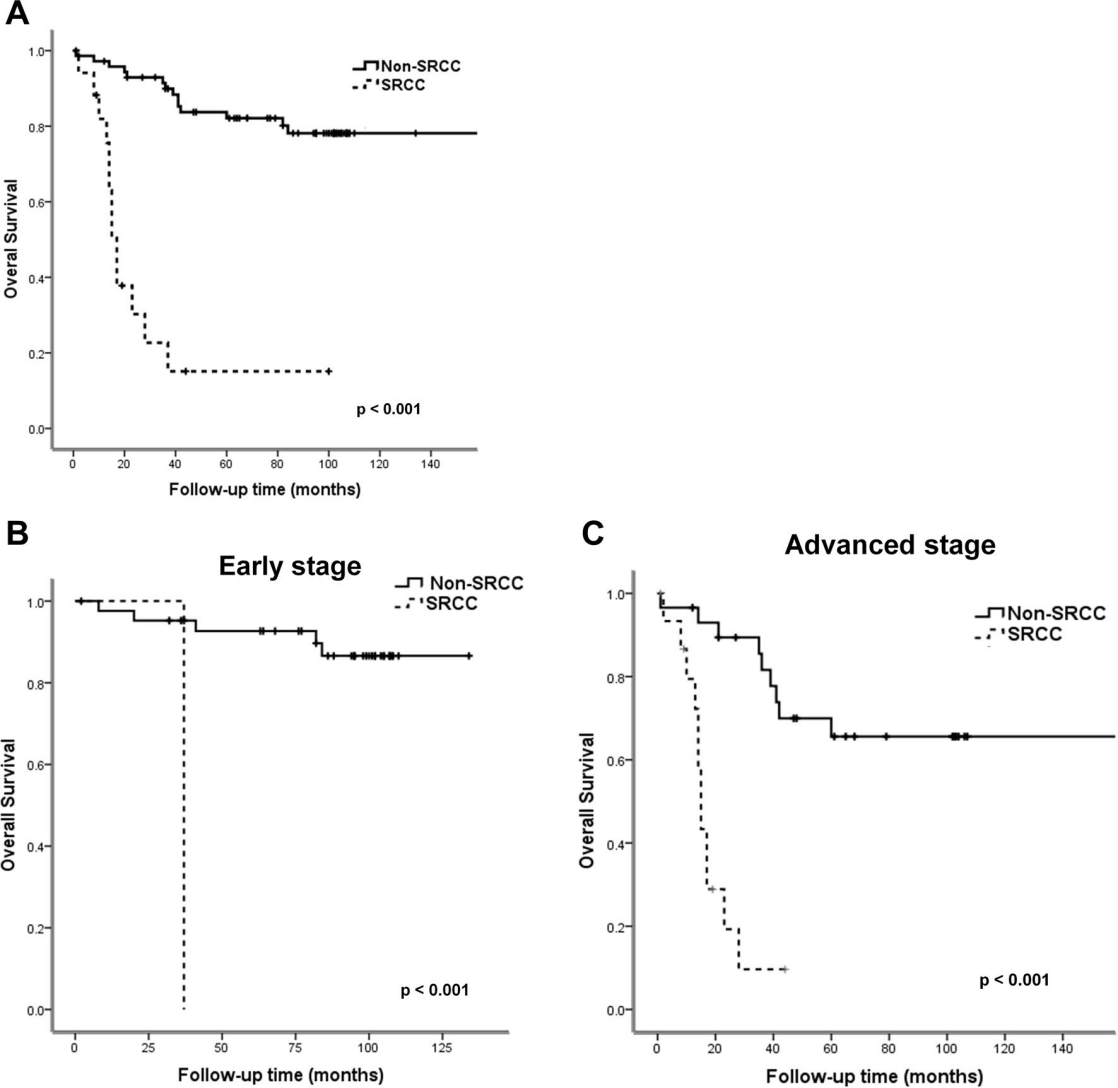
Kaplan–Meier estimated overall survival curves. **A** Overall survival of all patients with SRCC and Non-SRCC CRC. SRCC is associated with poor overall survival (log-rank *p* < 0.001). **B** Overall survival of patients with early stage SRCC and Non-SRCC CRC (log-rank *p* < 0.001). **C** Overall survival of patients with advanced stage SRCC and Non-SRCC CRC. (log-rank *p* < 0.001)

**Fig. 3:**
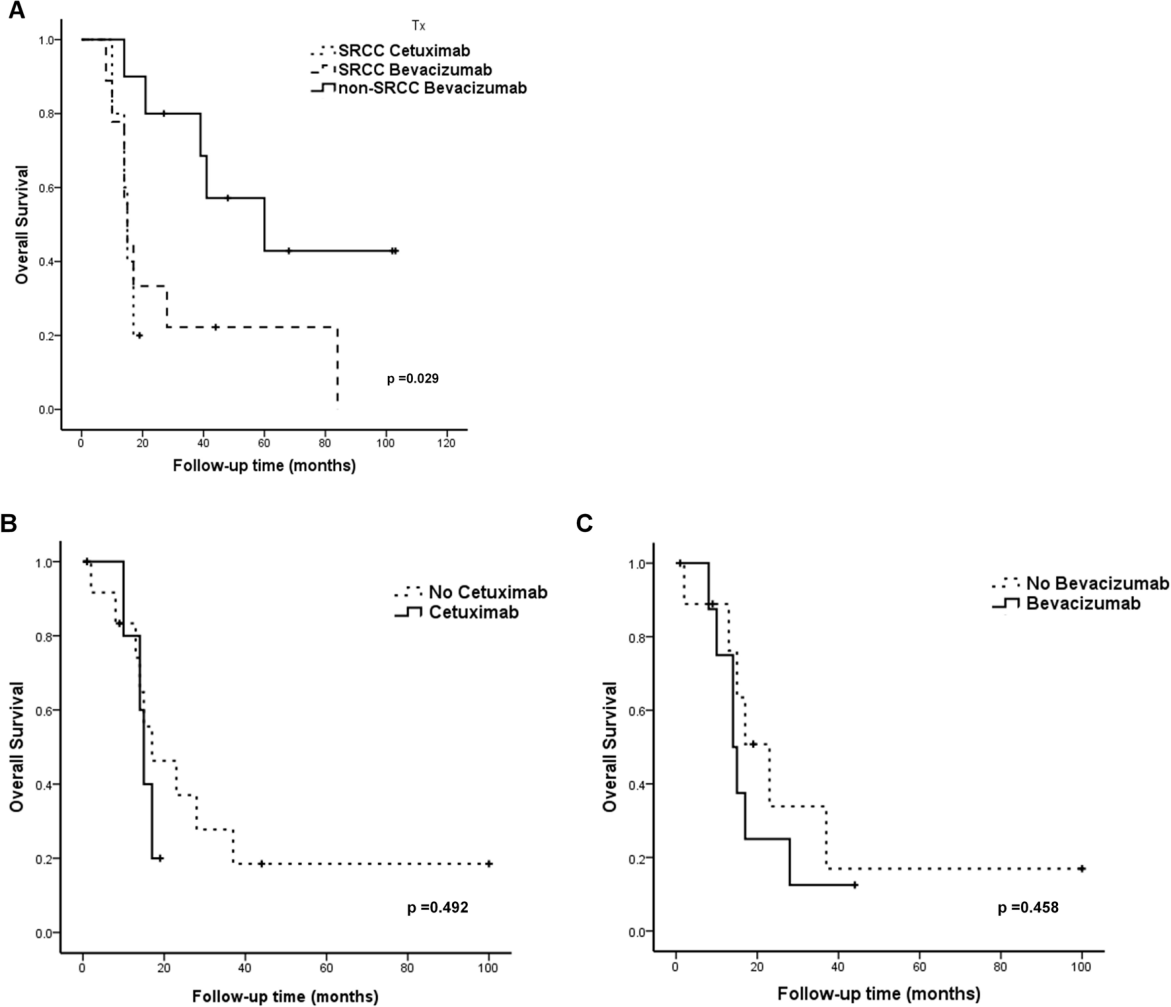
Kaplan–Meier estimated overall survival curves. **A** Overall survival of target therapy treated CRC patients. **B** Overall survival of SRCC patients received Ceuximab or not. **C** Overall survival of SRCC patients received Bevacizumab or not

The patients in the PSRCCR group had significantly poorer estimated overall survival than those in the Non-SRCC group (29.6 vs 162.7 months, log-rank *p* < 0.001, Fig. 2A). After stratification, the patients with PSRCCR still had significantly poorer estimated overall survival than did the patients with non-SRCC of early stage CRC ((37 vs. 122.8 months, log-rank *p* < 0.001, Fig. 2B) and advanced stage CRC (18 vs. 140.7 months, log-rank *p* < 0.001, Fig. 2C). Since majority of the patients were diagnosed at advanced stage, most of them received chemotherapy and target therapy. Target therapy prescribed in this study included Cetuximab and Bevacizumab, and all non-SRCC CRC patients received Bevacizumab. Non-SRCC patients still had better prognosis than PSRCCR patients no matter which target therapy they used (Fig. 3A). Target therapy treated or not didn’t improve the overall survival of PSRCCR patients (Fig. 3B, C).

### Factors associated with overall survival

In the univariable analysis of overall survival in all CRC patients, poor differentiation grade, lymphovascular invasion, advanced cancer stage, high CEA level and histological SRCC subtype were associated with increased mortality rates (all *p* < 0.05, Table 5). Further multivariate analysis with adjusted Cox proportional hazard model revealed that the CEA level (HR, 1.003; 95% CI, 1.000–1.005; *p* = 0.03) and histological SRCC subtype (HR, 8.333; 95% CI, 1.42–50; *p* = 0.005) were independent predictors of overall survival. The detail information of univariable and multivariate factor were listed in Table 5.

**Table 5:** Univariable and Multivariable Analysis of Overall Survival in colorectal cancer patients

	Univariable Analysis		Multivariable Analysis	
	HR (95% CI)	*P* value	HR (95% CI)	*P* value
Age	0.993 (0.969–1.018)	0.574		
Sex (male vs female)	1.091 (0.49–2.428)	0.832		
Grade (low vs high)	0.131 (0.057 vs 0.301)	< 0.001	0.998 (0.184–5.4)	0.998
Location (right vs left)	0.606 (0.281–1.306)	0.201		
Lymphovascular invasion (no vs yes)	0.265 (0.112–0.631)	0.003	0.359 (0.109–1.186)	0.093
Stage (early vs advanced)	0.194 (0.078–0.484)	< 0.001	0.599 (0.184–1.947)	0.394
CEA (ng/mL)	1.003 (1.002–1.005)	< 0.001	1.003 (1.000–1.005)	0.03
Subtype (SRCC vs non-SRCC)	10.64 (4.74–23.8)	< 0.001	8.333 (1.42–50)	0.005

The multivariate analysis only included the variables which P value < 0.05 in univariate analysis

HR, Hazard ratio, CI, confidence interval

## Discussion

Primary signet ring cell carcinoma of colon and rectum is a rare variant of CRC. The frequency of SRCC was no difference between Western and Eastern Countries. In this study, 18 of the 11,515 CRC patients were diagnosed with PSRCCR. It accounted for 0.16% of all primary CRCs. The percentage of PSRCCR was even lower in our study than those of previous studies which indicated 0.6%–2.7% [1, 7, 10, 11, 13].

Compared to non-SRCC patients, PSRCCR patients were younger. In Wang’ s study, they also reported PSRCCR is four times more prevalent among the young (ages < 40 years) than older adults (> 40 years) [14]. The underlying disease including hypertension, diabetes mellitus, viral hepatitis, coronary artery disease and chronic kidney disease were not significant different between PSRCCR and non-SRCC patients. Most (88.8%) PSRCCR patients were diagnosed at advanced stage as their initial presentations were non-specific such as abdominal fullness, pain and bowel habit change. The PSRCCR patients were also associated with low level of albumin and higher CEA level than non-SRCC patients. The high CEA level was related to 40% of SRCC patients diagnosed with stage IV.

Most of our patients’ CT finding (76.5%) presented with long segmental wall thickening rather than an intraluminal mass. In Kim’s study, they also reported the CT features of PSRCCR was long segmental (> 5 cm) concentric bowel wall thickening without an intraluminal mass, which resembles the inflammatory process [2]. 82.4% of tumor configuration were ulcerative or infiltrative type and only 17.6% were exophytic type in the PSRCCR group. However, in non-SRCC CRC group, infiltrative type only accounted for 4.2% and exophytic type for 47.2%. This is compatible with Messerini study [7] that revealed that infiltrative type was predominant in PSRCCR tumors (70.6% infiltrative type and 29.4% exophytic type). Most colorectal cancer cases (93.1%) were diagnosed by colonoscopic biopsy in control group, whereas 4 of SRCC patients (22.2%) ever received colonoscopic biopsy but failed to identify malignant cells. As 82% of PSRCCR were infiltrative or ulcerative type, it increased the difficulty to identify cancer cell by endoscopic biopsy and led to delay diagnosis. This also explained the reason that half of PSRCCR patients were diagnosed depended on direct operation for colon obstruction, bloody stool or malignant cells identified at non-colon site. Long segment colonic stenosis in young patients, also leading to the possibility of inflammatory bowel disease. Indeed, at least one of this PSRCCR cohort was treated as Crohn’s disease initially. Therefore, close monitoring the treatment effect and get histology diagnosis are important in the differential diagnosis.

Half of non-SRCC CRC patients underwent curative operation and had good prognosis. Most of PSRCCR patients were diagnosed at advanced stage and received combination therapy, whereas chemotherapy or target therapy both failed to improve survival. We also stratified and analyzed the treatment efficacy of bevacizumab or Cetuximab and showed poorer response in SRCC patients. Previously, SRCC has been shown to have fair response to chemotherapy and our results also confirmed this result. Furthermore, we showed that even with current available target therapy (anti-VEGF and/or anti-EGFR), the survival still could not be improved. Novel therapy, either chemotherapy or target therapy for PSRCCR patients, remains as an unmet need.

The estimated overall survival time of the PSRCCR patients (26.9 months) was much shorter than that of non-SRCC patients (162.7 months). SRCC subtype and elevated CEA were independent predictors of overall survival by using Cox proportional hazard regression model. Compared with non-SRCC patients, PSRCCR patients had higher risk of lymphovascular invasion, poor-differentiated carcinoma, visceral peritoneum invasion, lymph node and distant metastasis. This also indicated that the behavior of PSRCCR was more aggressive. According to Huang et al. [15] study, patients aged < 35 years had shorter cancer-specific survival compared with those aged > 35 years, and the 5-year cancer-specific survival rates were 31.1% and 54.9% in patients aged ≤ 35 and > 35 years, respectively. Five of the 18 patients in our series were aged < 35 years. The mean survival time was 20.6 months in this group. The survival time was not significantly different between these two groups. However, the results should be interpreted cautiously because of relatively few cases.

There were still limitations of this study. First, this was a retrospective observable study in single referral center. Second, the case number was small. As the PSRCCR is a very rare disease, it is difficult to collect many patients or perform a prospective study.

## Conclusion

Primary signet ring cell carcinoma of colon and rectum usually present as infiltrative or ulcerative configuration and is associated with poor differentiation, higher lymphovascular invasion and distant metastasis. Signet ring cell carcinoma is a strong predictor of poor overall survival. For young patients with colonic long segment stenosis and ulcerative/ infiltrative mucosa but endoscopic biopsy failed to identify malignant cells, PSRCC should be considered. Even with the progress in current chemotherapy and target therapy, they seemed to be with limited effect in improving the survial of PSRCCR patients and we still need to work out the novel therapy in order to improve the outcomes of these patients.

## Data Availability

The datasets used and/or analysed during the current study available from the corresponding author on reasonable request.

## Abbreviations

CD: Crohn’s disease
CRC: Colorectal cancer
IBD: Inflammatory bowel disease
NTUH: National Taiwan University Hospital
PSRCCR: Primary signet ring cell carcinoma of the colon and rectum
SRCC: Signet ring cell carcinoma
UC: Ulcerative colitis
